# Epigenetic effects of herbal medicine

**DOI:** 10.1186/s13148-023-01481-1

**Published:** 2023-05-13

**Authors:** Yu-Yao Wu, Yan-Ming Xu, Andy T. Y. Lau

**Affiliations:** Laboratory of Cancer Biology and Epigenetics, Department of Cell Biology and Genetics, Shantou University Medical College, Shantou, 515041 Guangdong People’s Republic of China

**Keywords:** Herbal medicine, Epigenetics, Cancer, Chronic diseases

## Abstract

Epigenetic memory is essential for life that governs the predefined functional features of cells. Recent evidence has indicated that the epigenetic modification provides a potential link to gene expression changes that may be involved in the development of various chronic diseases, and targeting the epigenome becomes a plausible method for treating diseases. Traditional herbal medicine has gradually entered the vision of researchers due to its low toxicity and its effectiveness in treating diseases. As a matter of fact, researchers found that the possessed epigenetic modification capacity of herbal medicine had the ability to combat the progression of the disease, such as various types of cancer, diabetes, inflammation, amnesia, liver fibrosis, asthma, and hypertension-induced renal injury. Studies on the epigenetic effects of herbal medicine will provide valuable insights into the molecular mechanisms of human diseases, which may lead to new therapeutic approaches and diagnoses. Thus, this review summarized the impact of herbal medicine and its bioactive components on disease epigenome as examples of how utilization of epigenetic plasticity could be useful as the basis for the future development of targeted therapies in chronic diseases.

## Background

Chronic diseases have now become a major health problem threatening to developing and developed countries, and if the situation is not effectively improved, the pandemic of chronic diseases will become a great burden to the global healthcare systems [1]. Sadly, most chronic diseases are difficult to cure, and patients can only take drugs to prevent the aggravation of the disease and delay the progress of the disease, such as cancer, coronary heart disease, diabetes and so on [2–4]. However, these drugs have more or less side effects, so understanding the pathogenesis of chronic diseases and finding appropriate therapeutic agents are urgently needed.

Epigenetic mechanisms play an important role in promoting the development of chronic diseases [5]. Epigenetic modifications mainly include DNA methylation, noncoding RNA, as well as histone modifications [6]. It has been found that noncoding RNA regulates gene transcription by inducing DNA methylation and histone modifications [7, 8]. DNA methylation and histone modifications are involved in many cellular processes and multiple human diseases [9]. Many substances can cause damage to the body by affecting the epigenetic state, resulting in the occurrence of chronic diseases. E-cigarettes ingredients (nicotine, tobacco-specific nitrosamines, volatile organic compounds, carbonyl compounds and toxic metals) can influence the occurrence of chronic bronchitis, facilitate cancer, neurodegeneration, etc., through DNA methylation, histone modifications or noncoding RNA expression [10]. Besides, epigenetic changes associated with cancer risk factors may play an important causal role in the development of cancer [11]. At the same time, disrupting the balance of epigenetic modifications within the body may lead to multiple pathologies, such as obesity and type 2 diabetes mellitus (T2D) [12]. Thus, a compromised epigenetic state plays a pivotal role in contracting various diseases, while the reversal of aberrant epigenetic modifications provides an exciting opportunity for the development of clinically relevant therapies [13].

Traditional Chinese Medicine (TCM) has a long history in China and TCM is an important category of complementary and alternative medicine, its use has increased in place in western countries over the past decade and the typical TCM therapies include acupuncture, herbal medicine and qigong exercises [14]. The three most common diseases of TCM users were tumors (33.2%), respiratory diseases (32.9%) and infectious diseases (8.86%), while the most commonly used TCM therapy is the Chinese herbal medicine and patients with comorbid diseases such as allergic rhinitis, indigestion, menstrual disorders, musculoskeletal system and connective tissue disorders tend to visit TCM clinics [15]. Studies have also indicated that herbal medicine can influence the progression of diseases through epigenetic modifications, including cancer, Alzheimer's disease, male infertility, etc. [16–18].

Understanding the regulation of the human epigenome by herbal medicine can help to elucidate the discovery of plant pharmacology and epigenetic drugs [19]. Therefore, this review mainly combines the relevant literature of nearly 10 years to discuss the epigenetic modification of chronic diseases through DNA and histone modifications by herbal medicine, in order to provide ideas for future disease research and treatment.

### Bioactive compounds from herbal medicine

Herbal medicine has been serving the Chinese people since ancient times and plays an important role in today's medical care [20]. According to a 1995 survey, there are 12,807 Chinese medicinal resources in China, including 11,146 medicinal plants [21]. TCM is mainly composed of botanical medicine (root, stem, leaf, and fruit) and mineral medicine. Because plant medicine accounts for the majority of TCM, TCM is also called herbal medicine. Although Western medicine has achieved remarkable results in the treatment of many diseases, the main challenges remain: infectious diseases that rapidly evolve to develop drug resistance to drugs, new diseases, especially new diseases caused by viruses, and ineffective long-term treatment of chronic and non-communicable diseases. TCM can provide complementary treatment based on personalized interventions to address the impact of disease on the whole body [22]. Most of the TCM preparations are oral preparations, such as decoction, pills, powder and other TCM dosage forms, as well as modern dosage forms such as granules, tablets and capsules. The oral preparations of TCM are the same as the chemical drug preparations containing one or more active ingredients, which first need to be absorbed through the gastrointestinal tract. TCM is rich in various components, leading to the complex absorption mechanism of drugs in the gastrointestinal tract, which is also one of the main differences between TCM and chemical drugs [23]. Although each herbal medicine contains hundreds or even thousands of components, only a few compounds can produce the drug and/or toxic effects [24]. Table 1 shows the active components with epigenetic modification effects as well as plants that are currently known to acquire these components. The active ingredients are rich in different herbs, which give these plants the characteristics to treat different diseases. Based on the efficacy of many bioactive compounds first discovered in herbal extracts, such as paclitaxel, camptothecin, and artemisinin, more people are accepting herbal medicine as potential sources of clinical drugs [25].

**Table 1:** Major active components with epigenetic modification effects from different herbal medicine

Active component	Chemical structure	Source plant		References
		Latin name	Common name	
Allicin		*Allium sativum*	Garlic	[26]
Baicalin		*Scutellaria baicalensis*	Chinese skullcap	[27]
Calebin-A		*Curcuma longa*	Turmeric	[28]
Cannabidiol		*Cannabis sativa*	Hemp	[29]
Cannabigerol		*Cannabis sativa*	Hemp	[29]
Cucurbitacin B		*Trichosanthes cucumerina*	Snake gourd	[30]
Curcumin		*Curcuma longa*	Turmeric	[31]
Curcumol		*Curcuma kwangsiensis*	Curcumae rhizoma	[32]
Esculetin		*Citrus limonia, Cortex fraxini, Ceratostigma willmottianum*	Lemon leaf, Ash bark, Chinese plumbago	[33]
Hesperetin		*Citrus *	Citrus fruits	[34]
Hydroxysafflor yellow A		*Flos carthami*	Safflower	[35]
Juglanin		*Polygonum aviculare*	Bianxu	[36]
Kazinol Q		–	Formosan plant	[37]
Kaempferol		*Cucurbita maxima,* *Daucus carota,* *Gingko biloba, Pinus densiflora,* *Angelicae decursiva,* etc	Pumpkin, Carrot, Gingko, Japanese red pine, etc	[38]
Luteolin		*Apium graveolens, Capsicum annuum, Perilla frutescens, Camellia sinensis*	Celery, Green pepper, Perilla leaf, Chamomile tea	[39]
Moringa isothiocyanate		*Moringa oleifera*	Drumstick tree	[40]
Naringenin		*Aurantii fructus*	Immature trifoliate-orange fruit	[41]
*N*-butylidenephthalide		*Angelica sinensis*	Danggui	[42]
Nimbolide		*Azadirachta indica*	Neem	[43]
Nordihydroguaiaretic acid		*Larrea tridentata*	Creosote bush	[44]
Oleanolic acid		*Oleaceae*	Oleaceae family plants	[45]
Osthole		*Cnidium monnieri*	Shechuangzi	[46]
Parthenolide		*Tanacetum parthenium*	Feverfew	[47]
Piperine		*Piper nigrum*	Black pepper	[48]
Sennoside A		*Rheum rhabarbarum*	Rhubarb	[49]
Silibinin		*Silybum marianum*	Milk thistle	[50]
Tanshinone I		*Salvia miltiorrhiza*	Danshen	[51]
Tetramethylpyrazine		*Chuanxiong rhizoma*	Chuanxiong	[52]
Triptolide		*Tripterygium wilfordii*	–	[53]
Valeric acid		*Valeriana officinalis*	Valerian	[54]
Zaluzanin D		*Gymnanthemum cass*	Vernonia arborea	[55]
Z-ligustilide		*Chuanxiong rhizoma*	Chuanxiong	[56]

### Epigenetic mechanism of herbal medicine

**Fig. 1:**
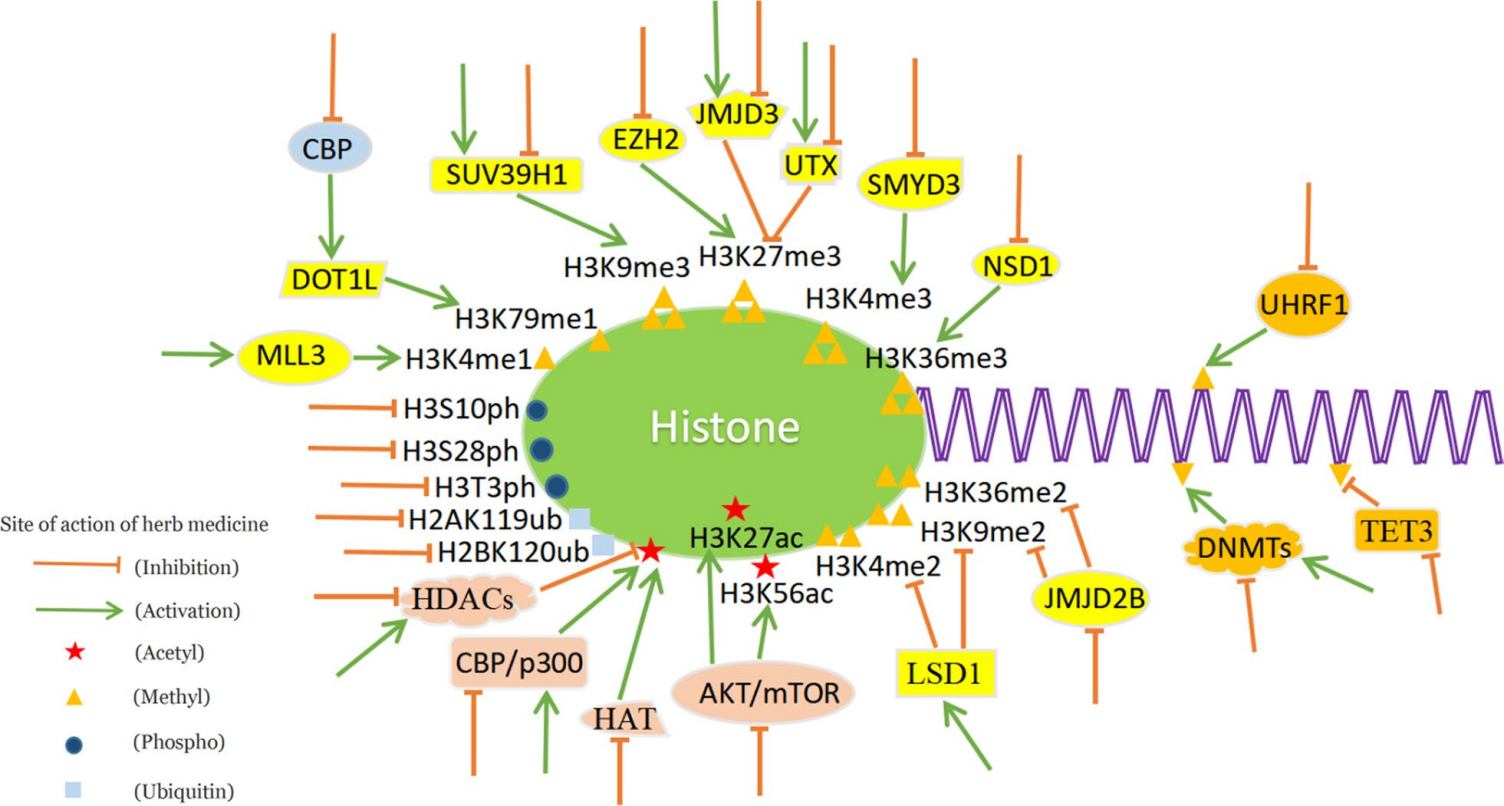
Epigenetic molecular targets of herbal medicine in cells. Herbal medicine can regulate histone methylation, histone acetylation and DNA methylation by affecting cellular factors directly, which are respectively located in the yellow, pink and orange patterns of the picture. Besides, herbal medicine can also affect histone ubiquitination and phosphorylation

DNA methyltransferases (DNMTs) and histone deacetylases (HDACs) are associated with the occurrence and progression of human malignancies, and DNMT and HDAC inhibitors are currently being explored as anticancer drugs in clinical trials [57, 58]. DNMTs can mediate specific DNA methyl transfer leading to epigenetic silencing of multiple genes [59]. HDACs catalyze the deacetylation of lysine residues in the N-terminal tail of histone proteins and regulate the expression of related genes [60]. Regulating the expression or activity of DNMT and HDAC is the most common way that herbal medicine and its bioactive components combating the disease through epigenetic regulation. In addition to these, as shown in Fig. 1, herbal medicine can also regulate the expression of related genes by affecting histone methylation, acetylation, phosphorylation, ubiquitination, as well as the demethylation modification of DNA.

### Brain tumor

There are about 120 types of brain tumors, about 45% of the primary brain tumors are glioma, and glioma or astrocytoma is one of the most common and aggressive brain tumors in children and adults [61]. For these cancers, very few effective treatment methods, even after active surgery, chemotherapy and radiotherapy, the patient survival rate is still very low [62].

Since 2014, Skala and Sitarek et al. have investigated the effects of Chinese herbal medicine on glioma, and initially they found that *Leonurus sibiricus* transgenic roots and *Rhaponticum carthamoides* transformed root are able to promote glioma cell apoptosis and inhibit their viability. Poly ADP-ribose polymerase 1 (PARP1) cleavage increasing *γ*H2A.X histone levels is necessary for the repair of DNA double-strand breaks and maintenance of genomic stability [63]. While Ubiquitin-like with plant homeodomain and ring-finger domains 1 (UHRF1) and DNMT1 are capable of epigenetic regulation of histone ubiquitination and DNA methylation [64]. Through further studies, they found that the cell-induced anticancer effects of *Leonurus sibiricus* extracts were associated with the number of γH2A.X and cleaved PARP1, and the level of UHRF1 and DNMT1 [65]. At the same time, *Rhaponticum carthamoides* extract can also trigger apoptosis in glioma cells by inducing DNA damage, PARP cleavage and epigenetic modification [66]. Topoisomerase II*β* (TopoII*β*) is a ribozyme that plays an important role in neuronal development. Yan et al. [52] conducted chromatin immunoprecipitation analysis and found that tetramethylpyrazine enhanced the recruitment of ac-H3 and ac-H4 in the promoter region of the *TopoIIβ* gene. Therefore, herbal medicine can also promote high TopoII*β* expression through epigenetic regulation and stimulate the neuronal differentiation of SH-SY5Y cells.

### Thoracic tumor

Thoracic malignancies include some of the most common and lethal cancers. It is expected that in the near future, the increase in cancer mortality is mainly related to smoking-induced lung cancer (including men and women), and female breast cancer [67]. In the 2008 study, tanshinone I showed the potential as an effective adjuvant in the treatment of human breast cancer, which effectively inhibited the proliferation of breast cancer cells MCF-7 and MDA-MB-231 while promoting its apoptosis [68]. Aurora A is a potential tumor marker, which is mainly localized to the spindle poles and the mitotic spindle, regulating the function of centrosomes, spindle bodies and kinetochores required for the normal progression of mitosis [69]. Inhibition of Aurora A directly reshaped the immune microenvironment by removing tumor-promoted myeloid cells and enriching anticancer T lymphocytes, which established a tumor-suppressive microenvironment and significantly promoted mammary tumor regression in mice [70]. In 2012, Gong et al. [71] showed that tanshinone I may downregulate *Aurora A* gene expression by reducing the ac-H3 associated with the primer 4-amplified area in the DNA promoter of the *Aurora A* gene, so inhibit the growth of breast cancer cells. In addition, herbal medicine can also affect the proliferation of breast cancer cells through the epigenetic regulation of matrix metalloproteinases (MMPs), a family of zinc-dependent endopeptidases that play a key role in cancer progression and metastasis [72]. The AKT/mTOR signaling pathway regulates the H3K27ac and H3K56ac, and Wu et al. [73] treated the cells with luteolin and found the levels of p-AKT and mTOR proteins were significantly reduced, thus increasing the overall occupancy levels of H3K27ac and H3K56ac in the MMP-2 and MMP-9 promoter regions, significantly inhibiting the expression of MMPs. At the same time, they also found luteolin increased the overall levels of H3K4me1 in MCF7-TamR cells, and decreased the overall levels of H3K4ac, which enhances H3K4me1 occupancy to the *Ras* gene family promoter and suppresses its expression [74]. Besides, oncogenes play an important role in tumor development. After treating the breast cancer cells with cucurbitacin B isolated from the traditional herbal medicine *Trichosanthes cucumerina*, Dittharot et al. [30] found that cucurbitacin B can upregulate DNMT1 and hypermethylation in c-Myc, cyclin D1 and survivin promoters, thereby downregulating the expression of all these oncogenes. Thus, cucurbitacin B has proven to be a potential cancer therapeutic, in part through the induction of hypermethylation and silencing of oncogenic activation. Herbal medicine is also able to epigenetically regulate the development of lung cancer. Lu et al. [75] have used Jinfukang (JFK), a clinical medicine usually used to treat lung cancer, to investigate whether the epigenetic modification is involved in its anticancer activity. The results showed that A549 cells treated for 48 h with JFK reduced the H3K4me3 modification levels of *SUSD2*, *PTN*, *GLIS2*, *CCND2*, *TM4SF4*, *BCL2A1*, *IL31RA*, *WISP2*, *TNFAIP6* and *TMEM158* genes. Besides, MYC and EGFR, two genes known to have high levels of H3K4me3 in A549 cells, also showed a significantly reduced degree of H3K4me3 expression after JFK treatment. From these studies, we can see the potential of herbal medicine for therapeutic use for thoracic cancer.

### Digestive system tumor

Digestive system cancer mainly consists of esophageal cancer, stomach cancer, small intestine cancer, colon–rectum cancer, liver, and pancreatic cancer. The incidence and mortality of digestive system cancer are very high, most of which are highly related to genetics and lifestyle [76]. In 2019, Li et al. [77] found that oleanolic acid, widely found in oleaceae family, inhibited the proliferation of human MKN-45 and SGC-7901 cells. Through further studies, in 2021, they identified epigenetic regulation of gastric cancer. Since immunotherapy through programmed cell death protein 1 (PD-1)/programmed cell death ligand 1 (PD-L1) blockade has shown benefits for gastric cancer, epigenetic DNA methylation critically modulates cancer immune checkpoints. They stimulated human gastric cancer MKN-45 cells with interleukin-1*β* (IL-1*β*) and significantly increased PD-L1 expression. After treating cells with oleanolic acid the IL-1*β*-increased DNA demethylase activity was abolished in MKN-45 cells, and oleanolic acid selectively reduced the expression of DNA demethylase tet methylcytosine dioxygenase 3 (TET3) induced by IL-1*β*, and overexpression of TET3 restored oleanolic acid-reduced PD-L1 expression. Their findings suggest the potential of oleanolic acid as an epigenetic modulator of immunotherapy or adjunctive therapy of gastric cancer [45]. In the same year, in mice, the epigenetic regulation of herbal medicine in gastric cancer was also found. One week after subcutaneous inoculation of MKN-45 cells in nude mice and gavage with hesperetin revealed that the levels of H3K79me2 and H3K79me3 were significantly reduced after hesperetin treatment. Moreover, the DOT1-like histone lysine methyltransferase (DOT1L) expression was also significantly decreased in vivo, and DOT1L is the only known H3K79 methyltransferase and can regulate cancer metastasis. To further confirm the effect of DOT1L on gastric cancer cell metastasis in vivo, MKN-45 cells were seeded into immunodeficient mice by tail vein injection. It was observed that downregulation of the *DOT1L* gene significantly inhibited the ability of MKN-45 cells to metastasize within the lung [78]. Thus, hesperetin targeting DOT1L may translate into future cancer treatment strategies. The *p16* gene belongs to the *INK4* gene family and consists of four members: p16 (INK4A), p15 (INK4B), p18 (INK4C), and p19 (INK4D). They all have common biological characteristics, namely cell growth suppression and tumor suppression, and p16 is the second most common tumor suppressor gene after p53 [79]. Supercritical CO_2_ extract of *Azadirachta indica* and nimbolide inhibited the expression of HDACs and DNMTs and significantly upregulated the acetylation levels of H3K9, H3K14, H3K18 and H3K27 in the p16 promoter region in HCT116 [80]. The p16 protein is inactivated in a variety of human cancers. Thus enhanced acetylation of the p16 promoter while reducing p16 methylation, both contribute to the restoration of *p16* gene expression, thereby affecting the expression of genes associated with cancer progression or repression that may be important targets for chemoprevention or therapy.

### Urogenital tumor

Although cancer drugs have been evolving in recent decades, the incidence and mortality of the most prevalent urogenital cancers have not been significantly reduced [81]. Prostate cancer is the second most common cancer in men [82]. Since 2013, Tamgue et al. [83] began epigenetic studies of triptolide on prostate cancer. They treated the prostate cancer cells with triptolide and found that triptolide significantly inhibited the proliferation of prostate cancer and was also able to reduce enhancer of zeste homolog 2 (EZH2) expression. EZH2 is the enzymatic catalytic subunit of the polycomb repressor complex 2 and can alter the expression of downstream target genes by H3K27me3 [84]. Besides, there is evidence that EZH2 plays an important role in cancer initiation, development, progression, metastasis, and drug resistance [85]. Then, in a 2017 study, Tamgue and Lei [86] found that although triptolide reduced EZH2 expression in PC-3 cells, the levels of H3K27me3 and histone H3 were increased. Therefore, other regulatory mechanisms may exist in PC-3 cells. Thus, they further found that the levels of mRNA and protein of UTX (also known as KDM6A) and histone demethylase Jumonji domain-containing 3 (JMJD3), which regulate H3K27me3 demethylation [87], decreased significantly in a dose- and time-dependent manners. Meanwhile, triptolide significantly increases the protein levels of SUV39H1, a histone methyltransferase that catalyzes the methylation of H3K9 [88], and related H3K9me3. In addition, they found that triptolide-induced deposition of H3K9me3 at the target gene promoters was highly SUV39H1-dependent and that inhibition of gene expression was partly mediated by enhancing the deposition of the H3K9me3 at gene promoter and inducing heterochromatin formation. These show the great clinical application value of triptolide. Besides, it has been found that components in herbal medicine can epigenetically regulate NRF2, a master regulator of many critical anti-oxidative stress defense genes in human prostate cancer [89], to regain their expression [90]. Lysine demethylase 1B (KDM1B) is a histone H3K4 demethylase required to establish maternal genomic imprints [91]. Lee et al. [92] found that *Oldenlandia diffusa* extract, by regulating KDM1B, effectively promoted the death of cisplatin-resistant ovarian cancer cells treated with cisplatin. However, the specific mechanism is still unclear and requires further experimental exploration.

### Blood tumor

Multiple myeloma is a clonal disease of long-lived plasma cells and is the second most common hematological cancer after non-Hodgkin's lymphoma. Malignant transformation of plasma cells gives them the ability to proliferate, causing harmful lesions to the patients [93]. The epigenetic studies on herbal medicine for myeloma were mainly conducted with triptolide by Wen et al. In 2010, using multiple myeloma cell line U266, it was found that triptolide dose-dependently reduced the genome-wide H3K4me3, H3K27me3, and H3K36me3, while also inhibiting SMYD3, EZH2 and nuclear receptor binding SET domain protein 1 (NSD1) expression [53]. Among them, SMYD3 is a SET domain-containing protein that has histone methyltransferase activity on histone H3K4, and SMYD3 is frequently overexpressed in different cancer cell types [94], which is associated with advanced stage and poor survival [95]. NSD1 is a bifunctional transcriptional regulatory protein able to participate in the regulation of mono- and dimethylation of H3K36, and targeting NSD1 may be a potential strategy for tumor therapy [96, 97]. Then, in 2012, by studying multiple myeloma (MM) cells, triptolide was demonstrated to decrease the overall H3K4me2 and H3K36me2 levels in a dose-dependent manner [98]. It also significantly increased the expression of lysine-specific histone demethylase 1 (LSD1), a nuclear histone demethylase [99], and decreased the JMJD2B expression which is a histone demethylase enzyme that regulates gene expression through demethylation of H3K9me3 and H3K36me2 [100, 101]. Lastly, in 2015, Wen et al. [102] treated KM3 cells with triptolide, and the results showed that triptolide can downregulate the expression of the proto-oncogenes c-Myc and VEGFA, a principal angiogenic factor essential for angiogenesis [103], by blocking the accumulation of H3K4me3 at its promoter. These results suggest that herbal medicine may have a strong effect against MM through epigenetic mechanisms. In addition, herbal medicine can regulate epigenetic regulation of leukemia and inhibit its development. Wang et al. [56] found that z-ligustilide increased the level of ac-H3 (K9/14) in HL-60 cells and enriched ac-H3 (K9/14) in the promoter region of Nur77 and NOR-1. At the same time, it significantly increased p300 acetyltransferase and decreased HDAC, including HDAC1 and HDAC4/5/7 and transfer-related protein 1, recruitment to the Nur77 promoter region. Z-ligustilide also enriched p-CREB in the NOR-1 promoter region, while HDAC1 and HDAC3 decreased in the NOR-1 promoter region. Thus, herbal medicine has strong potential in treating acute leukemia through epigenetic regulation.

### Other chronic diseases

Herbal medicine also plays an important role in the epigenetic regulation of the development of other diseases, such as diabetes, inflammation, liver fibrosis, obesity, amnesia, and so on. In a 2015 study, the function of esculetin in diabetes was identified by Kadakol et al. [104]. Posttranslational histone modifications (PTHMs) play a key role in the pathogenesis of diabetic complications [105]. They found that the treatment of hearts of IR and type 2 diabetic rats with esculetin reduced the originally elevated H3K4me2, H3K36me2, H3K79me2, H3S10ph, H3S28ph, H3T3ph, H3K27ac, H3K56ac, H2AK119ub, and H2BK120ub [106]. In 2017, it was demonstrated that esculetin treatment significantly improved vascular reactivity, increased eNos and decreased Vcam1 mRNA levels, and reduced collagen deposition in the rat thoracic aorta. At the same time, it can further improve vascular perturbation by reversing H2BK120ub to occupy the promoters of the *At1*, *At2*, *Tgfβ1*, and *Mcp1* genes [33]. In the same year, they also found that esculetin and telmisartan in combination therapy could improve type 2 diabetic cardiomyopathy by reversing H3, H2A, and H2B histone modifications [107]. These studies suggest that esculetin can be used as an advanced therapeutic agent, which may be partly attributed to its ability to reverse epigenetic alterations. Liver fibrosis occurs due to the long-term injury caused by the activated myofibroblast-mediated excessive wound healing response and the excessive scar deposition in the liver parenchyma. Although genetic effects are important, epigenetic mechanisms have been shown to orchestrate many aspects of liver fibrogenesis [108]. In 2020, by studying HSC-T6, sennoside A was demonstrated to reduce the expression of cyclin D1, CDK and c-myc and significantly inhibit the expression of p-AKT and p-ERK as well as *α*-smooth muscle actin and type I collagen alpha-1 protein levels. Meanwhile, sennoside A can directly bind to DNMT1 and inhibit its activity, thus significantly promoting phosphatase and tension homolog deleted on chromosome 10 (PTEN) expression in vitro [109]. However, the dynamic expression of PTEN in rat liver tissue was negatively correlated with liver fibrosis and activated hepatic stellate cells, and positively with the reversal of fibrosis and apoptotically activated hepatic stellate cells [110]. It thus follows that the epigenetic regulation of PTEN expression by sennoside A may be an effective new method for the treatment of liver fibrosis. In 2021, using the same cell line as with the animal model, it was shown that sennoside A consistently inhibits the expression of the liver fibrogenesis markers *α*-smooth muscle actin and type I collagen alpha-1 and suppresses the inflammatory response in vitro and in vivo. It can also promote SOCS1 expression in a DNMT1-dependent manner [49]. Besides, SOCS1 helps to protect against liver injury and fibrosis and may also protect against liver carcinogenesis [111].

All of the above information is integrated into Table 2.

**Table 2:** Herbal medicine-induced epigenetic alterations on chronic diseases

Year	Extract/component	Studying model/cell line	Epigenetic targets	Dose characteristics	Gene modified/epigenetic alterations	Outcome	References
Brain tumor							
2013	Curcumin	U87 and U251 cells	DNA	•30 μM curcumin for 4 days •10, 20, and 40 μM curcumin for 4 days	•Curcumin induces hypomethylation at 17 CpG sites on the RANK promoter •Curcumin inhibits the activity of DNMT1	Results in *RANK* gene activation in epigenetic modification in human glioblastoma cells	[31]
2014	Tetramethylpyrazine	SH-SY5Y cells	Histone	•80 μM tetramethylpyrazine for 3 or 5 days	•Tetramethylpyrazine enhances the recruitment of ac-H3 and ac-H4 to the *TopoIIβ* gene promoter region	Promotes SH-SY5Y cells to differentiate toward post-mitotic neurons	[52]
2018	*Leonurus sibiricus* transgenic roots extract	U87MG and grade IV glioma cells	DNA; Histone	•Treat the cells with the extraction for 24 h	•The extract downregulates the expression of UHRF1 and DNMT1	Influences epigenetic regulation	[65]
2018	*Rhaponticum carthamoides* transformed roots extract	U87MG and grade IV glioma cells	DNA; Histone	•Treat the cells with the extraction for 24 h	•The extract downregulates the expression of UHRF1 and DNMT1	Influences epigenetic regulation	[66]
2019	Calebin-A	STS26T, ST8814, T265, and S462-TY cells	Histone	•12.5 and 25 μM calebin-A •Calebin-A for 8 or 24 h	•Calebin-A decreases H3 histone acetylation •Reduces HAT activity	Epigenetic control of *survivin* and *hTERT* genes	[28]
Thoracic tumor							
2008	Nordihydroguaiaretic acid	T47D and RKO cells	DNA	•0–100 μM nordihydroguaiaretic acid for 72 or 144 h	•Nordihydroguaiaretic acid reverses p161NK4a CpG island hypermethylation	Induces cell cycle arrest in the G1 phase and a senescence-like state in cells	[112]
2008	Nordihydroguaiaretic acid	SKBR3 and MDA-MB-435 cells	DNA	•0–100 μM nordihydroguaiaretic acid for 7 days	•Nordihydroguaiaretic acid reverses methylation-silenced *E-cadherin* gene hypermethylation	Reactivates the expression of E-cadherin	[44]
2012	*Thymus serpyllum* extract	MDA-MB-231 cells	DNA; Histone	•250 and 500 μg/mL extract for 72 h	•The extract inhibits the DNMT and HDAC activities	Influences epigenetic regulation	[113]
2012	Tanshinone I	MCF-7 cells	Histone	•3 μM tanshinone I for 48 h	•Tanshinone I reduces ac-H3 associated with the primer 4-amplified area in *Aurora A* gene DNA promoter	Downregulates *Aurora A* gene expression	[71]
2014	Kazinol Q	MDA-MB-231 cells	DNA	•1, 2.5, 5, and 10 μM kazinol Q for 48 h	•Kazinol Q inhibits DNMT1 activity and reactivates the expression of a DNA methylation-silenced gene, *E-cadherin*	Inhibits cell viability	[37]
2016	Tien-Hsien Liquid	MCF-7 cells	DNA	•0–6 mg/mL Tien-Hsien Liquid for 72 h	•Tien-Hsien Liquid downregulates the protein level of DNMT1 and DNMT3a	Influences epigenetic regulation	[114]
2016	Jinfukang	A549 cells	Histone	•Treat the cells with Jinfukang for 48 h	•Jinfukang downregulates the H3K4me3 modification levels at *SUSD2*, *PTN*, *GLIS2*, *CCND2*, *MYC*, *EGFR*, and *TM4SF4* genes, whereas those at *BCL2A1*, *IL31RA*, *WISP2*, *TNFAIP6*, and *TMEM158* genes are upregulated	Inhibits cell proliferation	[75]
2017	Z-ligustilide	MDA-MB-231 cells	Histone	•50 μM Z-ligustilide for 72 h	•Z-ligustilide increases the enrichment of ac-H3 (K9/14) in the ER*α* promoter, and significantly reduces HDAC1, HDAC2, and HDAC4/5/7 at the ER*α* promoter	Induces cell cycle arrest and apoptosis	[115]
2018	Nimbolide	MDA-MB-231 and MCF-7 cells	Histone	•0–2.5 μM nimbolide for 48 h	•Nimbolide decreases HDAC2 and increases H3K27ac	Induces apoptosis, autophagy, cytoplasmic vacuolization and formation of autophagosomes	[43]
2019	Cucurbitacin B	MDA-MB-231, MCF-10A, and MCF-7 cells	DNA	•0–5 μM cucurbitacin B for 48 h	•Cucurbitacin upregulates DNMT1, as well as increases methylation in c-Myc, cyclin D1, and survivin promoters	Downregulates the expression of oncogenes, c-Myc, cyclin D1, and survivin	[30]
2020	Luteolin	MCF7-TamR cells	Histone	•10 and 20 μM luteolin	•Luteolin increases the expression of MLL3, increases the global level of H3K4me1, decreases the global level of H3K4ac, and increases the monomethylation level of H3K4 in the *Ras* gene enhancer and promoter region	Inactivates PI3K/AKT/mTOR pathway through repression of the *Ras* gene and thus causes apoptosis in tamoxifen-resistant breast cancer cells	[74]
2020	*Cotinus coggygria* Scop. extract	MCF-7 cells	DNA	•40.6 μg/mL *Cotinus coggygria* Scop. extract for 3, 24, 48, or 72 h	•Inhibits the expression of DNMT1 and DNMT3a	Causes S phase cell cycle arrest and triggers apoptosis, reduces colony formation, induces DNA damage, affects cellular thermodynamic parameters	[116]
2021	Valeric acid	MCF-7 cells	DNA; Histone	•0–10 μM valeric acid for 48 h	•Reduces HDAC activity and a global DNA hypomethylation	Decreases the breast cancer cell proliferation	[54]
2021	Luteolin	BT-20 and MDA-MB-231 cells	Histone	•10, 20 and 30 μM luteolin	•Luteolin decreases the level of H3K27ac and H3K56ac in the MMP-9 promoter region 2 and 3 in the BT-20 cells, and increases H3K27ac and H3K56ac on the MMP-2 and MMP-9 promoter region in MDA-MB-231 cells	Inhibits the proliferation and metastasis of androgen receptor-positive triple-negative breast cancer cells	[73]
Digestive system tumor							
2010	*N*-butylidenephthalide	HepG2 and J5 HCC cells	DNA	•50 μg/mL of *n*-butylidenephthalide for 48 h •25 μg/mL *n*-butylidenephthalide combined with 6.25–25 μM 1,3-bis(2-chloroethyl)-1-nitrosourea for 48 h	*•N*-butylidenephthalide downregulates the mRNA and protein level of MGMT •The combination treatment enhances methylation of the MGMT promoter	Inhibits the expression of MGMT and enhances apoptosis	[42]
2019	*Ginkgo biloba* extract	B6C3F1/N mice	DNA	•0, 200, 600, and 2000 mg/kg *Ginkgo biloba* extract, 5 days a week for 104 weeks	•The *Ginkgo biloba* extract-exposed affects the methylation of the hepatocellular carcinoma gene promoter	Influences epigenetic regulation	[117]
2019	Aged citrus peel extract	AML-12 cells	DNA; Histone	•0–100 μg/mL aged citrus peel extract for 72 h •50–1000 μg/mL aged citrus peel extract	•Aged citrus peel extract decreases the protein expression of HDAC8, DNMT1 and DNMT3a, enhances the protein expression of JMJD3 and UTX, and demethylates Nrf2 promoter •Inhibits CpG methyltransferase activity	Attenuates APAP-induced hepatic injury through the reactivation of NRF2 pathway in mouse AML-12 hepatocytes	[118]
2019	Supercritical CO_2_ extract of *Azadirachta indica*; Nimbolide	HCT116 and HT29	DNA; Histone	•40 and 75 μg/mL supercritical CO_2_ extract of *Azadirachta indica* for 48 or 96 h •5 and 10 μM nimbolide for 48 or 96 h	•Inhibits HDAC and DNMT activity and expression in both cell lines •Increases acetylation of H3K9, H3K14, H3K18, and H3K27 in the p16 promoter region and decreases methylation levels of H3K9me3 and H3K27me3 in HCT116 cells	Restores the expression p16	[80]
2020	Silibinin	RT4, 5637, and T24 cells	DNA	•50 μM silibinin for 24 h	•Silibinin induces global DNA hypomethylation	Influences epigenetic regulation	[50]
2020	Valeric acid	Hep3B, SNU-449, and HepG2 cells	Histone	•850 μM valeric acid for 24, 48, or 72 h	•Valeric acid inhibits HDAC1, HDAC2 and HDAC3 activity	Suppresses liver cancer development	[119]
2021	Oleanolic acid	MKN-45 cells	DNA	•0–40 μM oleanolic acid for 24 h	•Oleanolic acid selectively reduced the expression of TET3 in IL-1*β*-treated MKN-45 cells	Leads to DNA hypomethylation	[45]
2021	Hesperetin	Nude mice with MKN45 cells MKN-45 and HGC-27 cells	Histone	•Mice: 50 mg/kg hesperetin for 1 week •Cell: 0–100 μM hesperetin for 48 h	•Hesperetin decreases H3K79me2 and H3K79me3 levels in mice •Inhibits H3K79 methylation and reduces the abundance of Dot1L protein in the cells	Decreases the mobility of gastric cancer cells and inhibits the abundance of DOT1L	[78]
2021	Curcumol	HepG2 and SMMC-7721 cells	Histone	•60 μM curcumol for 48 h	•Curcumol increases the expression of H3K27me3 and H3K9me3 in HepG2 cells, decreases H3K27me3 expression in SMMC-7721 cells, and downregulates EZH2	Downregulates lncRNA Hotair in turn downregulated EZH2, thereby disrupting trimethylation of H3K9 and H3K27 specifically catalyzed by EZH2, and regulating histone modification to inhibit tumor growth and metastasis	[32]
2022	Baicalin	BALB/c nude mouse and HepG2 cells	DNA	•Mice: 20 or 50 mg/kg baicalin for 28 days •Cell: 50 mg/L baicalin for 24 h	•Baicalin downregulates the m6A/A, SAM/SAH, and m6A (2854) levels of HKDC1 in the tumor tissue of the BALB/c mice •Downregulates the total DNA 5mC and RNA m6A levels, upregulates SAM/SAH, and suppresses the RNA m6A (2854) of HKDC1 in HepG2 cells	Inhibits the progression of T2D-induced liver tumors by regulating the HKDC1/JAK2/STAT1/caspase-3 pathway	[27]
2022	*Phyllanthus debilis* methanolic extract	HT-29 cells	DNA	•0.1 mg/mL *Phyllanthus debilis* methanolic extract for 24 h	*•Phyllanthus debilis* extract increases the Alu DNA methylation and LINE-1 methylation	Anticancer effects	[120]
Urogenital tumor							
2012	Trichosanthin	HeLa and CaSki cells	DNA	•0, 20, 40, and 80 μg/mL for 48 h	•Trichosanthin inhibits DNMT1 enzyme activity and DNMT1 expression	Induces gene demethylation of both HeLa and CaSki cells	[121]
2012	Triptolide	Du145	Histone	•0, 25, 50, 100 nM triptolide for 24 h	•Triptolide decreases histone H3K27me3 methylation and downregulates EZH2	Influences epigenetic regulation	[122]
2013	Z-Ligustilide; Radix Angelicae Sinensis	TRAMP C1 cells	DNA	•50 µM Z-Ligustilide or 8.5 µg/mL Radix Angelicae Sinensis for 3 days	•Z-Ligustilide or Radix Angelicae Sinensis treatment reduces the methylation levels of the first five CpG of the NRF2 promoter and inhibits DNA methyltransferase in vitro	Results in the re-expression of NRF2 and NRF2 target genes	[90]
2013	Triptolide	PC-3 cells	Histone	•0–1 µM triptolide for 24 h	•Triptolide decreases the expression of EZH2	Results in increased mRNA levels of target genes (ADRB2, CDH1, CDKN2A, and DAB2IP), and decreased mRNA levels of gene (cyclinD1)	[83]
2014	Zyflamend	CWR22Rv1 cells	Histone	•200 µg/mL zyflamend for 0–60 min •200 μg/ml zyflamend for 24 h	•Zyflamend downregulates the expression of all class I and II HDACs, and upregulates the histone acetyltransferase complex CBP/ p300 •Increases histone 3 acetylation	Promotes the increased expression of the tumor suppressor *p21* gene	[123]
2015	Allicin	MIA PaCa-2 cells	Histone	•100 and 200 µM allicin for 24 h	•Allicin reduces the level of H3K9me, and increases the level of H3S10ph and H3K14ac	Modulates apoptosis and represses gene expression	[26]
2017	Triptolide	PC-3 cells	Histone	•0–100 nM triptolide for 24 h or 100 nM triptolide for 0–24 h	•Triptolide enhances H3K27me3 levels by downregulating JMJD3 and UTX and enhances H3K9me3 level through upregulation of SUV39H1	Influences epigenetic regulation	[86]
2019	*Oldenlandia diffusa* extract	A2780cis cells	Histone	•40 µg/mL and 160 µg/mL for 48 h	*•Oldenlandia diffusa* extract downregulates the epigenetic regulator KDM1B	Overcomes resistance to cisplatin in CRC by modulating epigenetic regulation	[92]
Blood tumor							
2010	Triptolide	U266 cells	Histone	•20, 80, and 160 nM triptolide for 48 h •20, 80, and 160 nM triptolide for 24 h	•Triptolide decreases the expression of histone H3K4me3, H3K27me3 and H3K36me3 •Decreases histone methyltransferases SMYD3, EZH2 and NSD1	Induces epigenetic alterations	[53]
2010	Triptolide	RPMI8226 cells	Histone	•0–160 nM triptolide for 48 h	•Triptolide decreases histone H3K9me3 and H3K27me3 and downregulates histone methyltransferase SUV39H1 and EZH2	Induces epigenetic alterations by regulating histone lysine methylation	[124]
2011	Tien-Hsien Liquid	NB4 cells	DNA	•0–3 mg/mL Tien-Hsien Liquid for 72 h	•Tien-Hsien Liquid downregulates DNMT1	Influences epigenetic regulation	[125]
2012	Triptolide	RPMI8226 cells	Histone	•50, 100, and 150 nM triptolide for 48 h	•Triptolide suppresses the expression of H3K4me2, H3K9me2 and H3K36me2 and alters the expression of histone demethylase LSD1 and JMJD2B	Restores epigenetic changes by regulating the histone demethylases LSD1 and JMJD2B	[98]
2015	Triptolide	KM3 cells	Histone	•0–160 nM triptolide for 48 h	•Triptolide blocks the accumulation of H3K4me3 on c-Myc and VEGFA promoters	Decreases the expression of *c-Myc* and *VEGFA* genes	[102]
2016	*Acanthopanax senticosus*	HL-60 and HL60/ADM cells	Histone	•100 μg/mL *Acanthopanax senticosus* for 6 h •100 μg/mL *Acanthopanax senticosus* for 0–24 h	•*Acanthopanax senticosus* decreases HDAC enzyme activity •Increases histone ac-H3	Induces apoptosis of leukemia cells, cell cycle arrest, and FasL expression by promoting histone H3 acetylation	[126]
2020	Zaluzanin D	PMA differentiated human monocytic THP-1 cells	DNA	•0.35 mM zaluzanin D for 24 h	•Zaluzanin D reduces hypomethylation of the *MMP9* gene promoter region caused by PMA activation	Influences the epigenetic machinery	[55]
2021	Z-ligustilide	Acute myeloid leukemia cells	Histone	•50 μM Z-ligustilide for 1 h •50 μM Z-ligustilide for 6 h	•Z-ligustilide increases the enrichment of ac-H3 (K9/14) in the Nur77 and NOR-1 promoters •Increases p300 acetyltransferase and decreases HDACs, including HDAC1 and HDAC4/5/7 and MTA1, recruitment to the Nur77 promoter region	Restores the expression of both Nur77 and NOR-1	[56]
Other chronic diseases							
2012	Parthenolide	JB6P + cells	Histone	•5 and 10 μM parthenolide for 24 h	•Parthenolide inhibits HDAC1 and increases dimethylation level of H3 (K9/K4) on p21 and cyclin D1 promoters	Epigenetically modulates p21 and cyclin D1 expression	[47]
2019	Moringa isothiocyanate	JB6P + cells	DNA	•2.5 μM Moringa isothiocyanate and/or 10 ng/mL 12-O-tetradecanoylphorbol-13-acetate for 5 days	•Moringa isothiocyanate reverses methylation changes in those genes (hyper- or hypomethylation) that occur in response to TPA	Affects the progression of skin carcinogenesis	[40]
2015	Esculetin	Type 2 diabetic rats	Histone	•50 and 100 mg/kg/day esculetin for 2 weeks	•Inhibits H3K4me2, H3K36me2, H3K79me2, H3S10ph, H3S28ph, H3T3ph, H3K27ac and H3K56ac, and decreases H2AK119ub and H2BK120ub in hearts of IR and type 2 diabetic rats	Restores normal levels of allowed PTHMs and H2A/H2Bub in the hearts of IR and diabetic hearts	[106]
2017	Esculetin	Type 2 diabetic rats	Histone	•50 and 100 mg/kg/day esculetin for 2 weeks	•Esculetin reverses the modification in H2BK120ub and decreases the mRNA levels of Usp16 and Usp22	Intervenes H2Bub system	[33]
2017	Esculetin	Type 2 diabetic rats	Histone	•50 mg/kg/day esculetin for 6 weeks	•Esculetin reduces H3K9ac, H2AK119ub and H2BK120ub level	Influences epigenetic regulation	[107]
2021	Naringenin and Hesperetin	INS-1 cells and C57BLKS/Lepr^db^ mice	Histone	•Cell: 100 μM naringenin or hesperetin for 24 h •Mice: 50 mg/kg/day naringenin or 50 mg/kg/day hesperetin for 6 weeks	•Naringenin and hesperetin suppress the acetylation of H3K18 and H3K27 and inhibit the activity of p300, and hesperetin suppresses H3K27 acetylation in the transcriptional regulatory region of *Txnip gene* in INS-1 cells •Inhibit the acetylation of H3K18 and H3K27 in the islets of the C57BLKS/Lepr^db^ mouse	Reduces the expression of TXNIP	[41]
2020	Hesperetin	RAW 264.7 cells	Histone	•100 μM hesperetin overnight	•Suppresses the acetylation of RelA/p65 by inducing SIRT1 expression	Reduces NF-κB activity	[127]
2022	Kaempferol	3T3-L1 cells	Histone	•100 μM kaempferol	•Kaempferol decreases H3K27me3 deposition in the promoter region of *Adipoq*, *Fabp4*, and *Lpl* genes	Suppresses the expression of PPAR*γ* target genes (*Adipoq*, *Fabp4*, and *Lpl*)	[38]
2020	*Bacopa monniera* extraction	Male Swiss albino mice	DNA; Histone	•120 mg/kg *Bacopa monniera* extract	•Reduces the expression of HDACs, and decreases the activity of DNMT and HDAC enzyme and global DNA methylation	Reverses epigenetic changes in scopolamine induced amnesia as well as able to recover levels of synaptic proteins	[128]
2019	Piperine	3T3-L1 cells	Histone	•50 μM for piperine 8 days	•Piperine decreases the enrichment of H3K27me3 PPAR*γ*, and H3K9ac, and increase EZH2	Augments the expression of Ezh2-associated lipolytic genes	[48]
2021	*Sophora flavescens* (SF)-F2	PBMCs	DNA	•22.5 μg/mL *Sophora flavescens* (SF)-F2 for 3 days	•*Sophora flavescens* (SF)-F2 combination with dexamethasone downregulates the Foxp3 promoter methylation at CpG	Counteracts dexamethasone-induced immuno-suppression	[129]
2021	Hydroxysafflor yellow A	hBMSCs	Histone	•10 μM hydroxysafflor yellow A for 48 h	•Hydroxysafflor yellow A increases the protein level of KDM7A and decreases the occupancy of H3K27me2 on beta-catenin promoter	Increases *β*-catenin expression	[35]
2020	Astragalus polysaccharide	Specific pathogen-free Female Sprague–Dawley rats	DNA	•150 mg/kg/day astragalus polysaccharide for 8 weeks	•Astragalus polysaccharide alters the DNA methylation group of the colon epithelium and induces promoter DNA methylation changes in genes involved in calcium homeostasis, osteocast/osteoblast balance, Wnt signaling, and hormone-related processes	Influences epigenetic regulation	[130]
2011	Multiglycosides of Tripterygium wilfordii Hook f extract	68 days male mice	Histone	•7.5–22.5 mg/kg/day multiglycosides of Tripterygium wilfordii Hook f extract for 40 days	•Reduces the dimethylation levels of histone H3K9 in germ cells	Inhibits the process of spermatogenesis	[131]
2020	Qian Yang Yu Yin Granule	The renal damage model of spontaneously hypertensive rats HEK293T cells	DNA; Histone	•Mice: 2.1 or 8.4 g/kg/d Qian Yang Yu Yin Granule for 8 weeks •Cell: 0.5 or 10 μg/mL	•Qian Yang Yu Yin Granule suppresses protein expression of NNMT and ac-cortactin and increased protein expression of H3K4me3 •Inhibits the production of NNMT and SAH mRNA, and promotes the production of SAM and SIRT1, and upregulates DNA methylation	Protects against hypertension-induced renal injury in spontaneously hypertensive rats and inhibited cells proliferation induced by Ang II	[132]
2021	Juglanin	High fat diet-fed mice	Histone	•7.5–30 mg/kg juglanin for 16 weeks	•Juglanin suppresses the expression of HDAC3 from mRNA and protein levels	Suppresses the activation of NF-κB/HDAC3 signaling in kidney of HFD-challenged mice	[36]
2012	Yang-Gan-Wan	Hepatic stellate cells isolated from C57Bl/6 and Coll-GFP mice	Histone	•Yang-Gan-Wan for 7 days	•Yang-Gan-Wan suppresses the expression of PRC2 components, EZH2, SUZ12, and EED, and increases H3K4me2 and H3ac at the PPARγ promoter locus	Prevents and reverses hepatic stellate cell activation	[133]
2020	Sennoside A	HSC-T6 cells	DNA	•10 μM sennoside A for 48 h	•Sennoside A blunts the activity of DNMT1 in TGF-*β*1-treated HSC-T6 cells	Inhibits activation and proliferation of HSC-T6 cells by targeting DNMT1	[109]
2021	Sennoside A	RAW264.7 cells	DNA	•Sennoside A 20 nM for 24 h	•Sennoside A decreases the activity of DNMT1 in LPS-treated RAW264.7 cells and inhibits SOCS1 hypermethylation mediated by DNMT1	Enhances SOCS1 expression	[49]
2014	Luteolin	THP-1 cells	Histone	•3, 6 and 10 μM luteolin for 48 h	•Luteolin downregulates HAT activity, upregulates HDAC activity and decreases the levels of acetyl CBP/p300 in high-glucose conditions	Affects NF-κB and p65 activation and interaction between p300 and NF-κB under hyperglycemic conditions in monocytes	[39]
2017	Osthole	PDLSCs	Histone	•10 μM osthole for 7 days	•Osthole increases the expression of KAT5, MOZ, MORF and ELP3, and increases the level of acetylation of H3K9 and H3K14	Reverses defective osteogenesis of P-PDLSCs	[46]
2013	Cannabidiol and cannabigerol	HaCaT cells	DNA	•0.5 μM cannabidiol or 0.5 μM cannabigerol for 5 days	•Cannabidiol and cannabigerol enhance DNMT1 expression	Increase global DNA methylation levels and decrease the expression of all the genes examined in the differentiated HaCaT cells by increasing the DNA methylation of the *keratin 10* gene	[29]
2021	Cannabidiol, luteolin, and piceatannol	CPEK cells	DNA	•10 μM cannabidiol, 25 μM luteolin, and 25 μM piceatannol for 8 h	•Increase the percentage of methylation in ccl2 CpG sites	Manage chronic inflammation through nutraceuticals that modulate DNA methylation	[134]
2022	Cooked rhubarb	Young male Sprague–Dawley rats	DNA	•3 g/kg/day cooked rhubarb for 8 weeks	•Regulates the level of DNA methylation and expression of *IL-1α* and *IL-10* genes	Reduces pathological tissue damage caused by chronic alcohol exposure	[135]

## Conclusions

Herbal medicine has a strong capacity to regulate the occurrence and progression of chronic diseases through epigenetics. In tumors, herbal medicine can regulate the expression of tumor-related genes by influencing the methylation and acetylation of histones at the gene promoter or enhancer by controlling the expression of CBP, SUV39H1, EZH2, JMJD3, UTX, NSD1, etc. At the same time, herbal medicine can also regulate the methylation of DNA by affecting the expression of TET3, UHRF1, DNMTs, etc. In epigenetic studies of other diseases, herbal medicine shows the great potential of clinical applications in treating amnesia, allergic asthma, diabetes, inflammation, and liver fibrosis. However, the study of the specific mechanism is still quite limited. In order to provide more comprehensive information on the epigenetic impact of herbal medicine on human diseases and to fully exploit its potential in the clinic, further well-designed in vivo studies should be conducted.

## Data Availability

Not applicable.
